# A New Tooth Crown

**Published:** 1888-02

**Authors:** John B. Parker

**Affiliations:** Grand Rapids, Mich.


### CORRESPONDENCE.

### A New Tooth Crown.

EDITOR OF REGISTER,

*Dear Sir:*—No doubt my long familiarity with the new tooth makes it seem strange to me that anyone should not comprehend it, and all its signifies, at the first glance.

First and foremost it has no resemblance to the old "plate tooth backed up," and to *no other form of tooth ever used* for plate or bridge work. It is an entirely new departure in porcelain teeth.

As you are aware, porcelain teeth have heretofore consisted of a single piece; a crown and body ready for a thin backing as in the case of the common plate tooth, or of a "shell" or "facing" to which the crown, made of gold, has to be attached.

In the case of the old plate tooth with a packing on one side of the tooth, there were elements of weakness that made it absolutely unreliable and useless for bridge work, as every dentist who has any experience with it knows. In the case of the crown of gold mounted on the "shell" or "facing," the conspicuousness of the gold, its unfitness for a grinding surface, its cost in precious metal, and the time consumed in getting it ready for the mouth, present a list of objections that are formidable, to say the least, *if* they can be avoided by a new device.

The objections to both these teeth--the old plate tooth and the shell--the new tooth I sent you entirely removes. In the case of the old plate tooth, the entire weight of the bite comes on the pins, and they pull out or break, or break the tooth, in bridge work, nearly always in a short time, and in plate work, frequently.

In the case of the gold capped "shell," the constant pressure of mastication gradually forces the gold covering against the shell and breaks *that* from its pins also, making an additional objection to its use. The new tooth obviates all these objections.

First: It will not break under the strain of mastication; Second, it presents a good grinding surface; Third, it is more artistic--avoiding the glitter of metallic surfaces; Fourth, it can be furnished for less than it costs the dentist to put a gold top on a "shell"; (the only tooth now in use that has any service in bridge work) and fifth, it is as convenient to use, as ready to be adjusted to bridge work without trouble or delay, as is the rubber tooth to rubber work.

The new tooth is made in two pieces: a shell and a crown; both provided with pins, and attached to a common support, which is a "backing" to the "shell," and a *foundation* to the crown as well.

The pins can't break, or stretch, or pull out of the crown, because the bite is always towards their ends. The shell can't break, because there is first, a metal covering which protects it from absolute contact with the crown, and second, because the pressure on the crown is chiefly borne by the *foundation* of the crown.

To sum up--what is new in this tooth is a combination of two separate pieces of porcelain attached to a common support, said pieces of porcelain set at substantially right angles to each other, so that the pressure of mastication shall fall in the direction of the greatest strength of the tooth, instead, as is the case with the common plate tooth backed up, in the direction of its greatest weakness.

Put your old plate tooth with the gold backing *underneath* the side of the tooth that is bitten on—viz, the backing next the gum, and the patient can never break it with pressure of the jaws. Set it up as is always done and in bridge work the weakest of them will chew it off its backing in time. As I have tried to make you see, the new tooth is elegant in appearance in the mouth, is easily soldered up by bridge workers, a better masticating tooth than the gold capped shell, and can be put into service successfully when every other porcelain tooth is a failure. In a word it is the *only* porcelain tooth for bridge work.

Yours truly,

Grand Rapids, Mich. JOHN B. PARKER.

